# Social workers’ views and experiences of self-care practices: a qualitative interview study

**DOI:** 10.3389/fpubh.2025.1585900

**Published:** 2025-03-27

**Authors:** Miao Jian, Pearse Mccusker, Mary Mitchell, Autumn Roesch-Marsh, Sarah Rose, Lora Petrova

**Affiliations:** ^1^Department of Social Work & Social Policy, School of Sociology, Nankai University, Tianjin, China; ^2^School of Social and Political Science, The University of Edinburgh, Edinburgh, Scotland, United Kingdom; ^3^School of Health and Social Care, Edinburgh Napier University, Edinburgh, Scotland, United Kingdom

**Keywords:** social worker, vulnerability, self-care, experience, workplace wellbeing, mental health

## Abstract

Self-care is increasingly advocated as necessary for improving social workers’ wellbeing. However, it remains a contested term, with limited understanding of social workers’ views and experiences of what it constitutes in practice. This qualitative study employed semi-structured interviews with nine social workers from three local authorities in Scotland. Informed by vulnerability theory, a six-phase thematic analysis was applied to explore social workers’ views and experiences of self-care practices. Three key themes emerged: (1) understanding and conceptualizing self-care, illustrating practitioners’ perceptions of self-care as individualized, multifaceted strategies aimed at both personal wellbeing and professional efficacy, with heightened awareness since COVID-19; (2) the implementation paradox, highlighting fundamental tensions between acknowledging professional vulnerability and managing organizational demands, workload pressures, and insufficient institutional support; and (3) toward sustainable self-care practice, identifying pathways through deliberate individual practices, organizational support, educational preparation, and culturally-sensitive policies. Public health policymakers and healthcare organizations should prioritize structural reforms to enhance workforce resilience, thereby improving service quality, practitioner wellbeing, and overall public health outcomes.

## Introduction

Professional vulnerability has emerged as a crucial area of study in mental health research, with social workers representing one of the most significant cases due to their direct engagement with complex societal problems, from poverty and inequality to maintaining health and human wellbeing (1). As helping professionals, social workers are consistently expected to act effectively for people in need (2, 3). However, the challenges arising from working conditions, including high case-loads, low job control, and ‘moral distress’ (where social work practice conflicts with values), exacerbated by resource constraints, neo-liberal organizational/management approaches, and complex multi-disciplinary working contexts, can precipitate vulnerability regarding work-related stress and negative self-perceptions of capability (4–6). If not well addressed, these challenges and risks can lead to turnover intention or other problems that impact the wellbeing of social workers and, consequently, the quality of service provided (7).

Self-care practices have emerged as a crucial strategy for addressing these professional vulnerabilities and supporting mental health among social workers. These practices have been recognized as effective and are recommended for social workers to help mitigate the negative impacts of working with traumatized populations, fostering self-compassion to combat fatigue in high-stress environments (8–10). Generally, self-care is viewed as the intentional practice of engaging in activities that promote one’s holistic health and wellbeing while also ensuring the effective and appropriate use of the self in their professional roles (11). The related practices involve various activities, including physical care, psychological care, emotional care, mental care, and occupational care (12). Physical care involves improving practitioners’ physical condition, including through exercise, a balanced diet, and adequate sleep. Psychological care includes enhancing self-awareness and decision-making through therapy, journaling, and reading. Emotional care focuses on supporting emotional equilibrium through positive behaviors like intimacy, self-encouragement, and laughter. Spiritual care involves finding meaning in life through practices such as meditation. Occupational care entails using professional resources and skills to enhance achievements and career satisfaction, such as participating in professional training, setting appropriate boundaries in relation to the professional social worker role, and being provided with effective guidance from supervisors, particularly when encountering difficulties. Ideally, these activities would be consistently and intentionally integrated into practitioners’ routines (13–15). A recent literature review has sought to add conceptual clarity to how self-care is defined in social work and, particularly, how it may be framed in ways that align with the profession’s core value of social justice and support anti-oppressive practice (16).

There has been a notable increase in the literature exploring self-care within social work, and the research evidence indicates that it can be effective in improving social workers’ wellbeing, for example, by bolstering their intrinsic motivations and abilities to address their personal needs (10, 14). Adopting self-care behaviors has also been shown to help buffer compassion fatigue, turnover intention, and secondary trauma (8) while improving social workers’ job satisfaction and enhancing their resilience (11, 17, 18). The COVID-19 pandemic has heightened recognition of the necessity for mental health support and self-care among social workers (19), emphasizing the importance of self-awareness and practical approaches to personal and professional wellbeing.

With the rapid expansion in the number of social workers across the world (20–22), the need for social workers to improve wellbeing in challenging economic contexts is also increasing. Researchers have provided some evidence-based studies to indicate for reference in social workers’ practices (10, 23). Existing research and evaluations on self-care interventions have been concentrated in countries like the United States and the United Kingdom (24, 25). Educational training programs developed by social work authorities or incorporated into higher education curricula are increasingly discussed and used in these regions, mostly aimed at equipping practitioners and students with the foundational knowledge of mental health issues and essential self-care skills (23, 26–28). Mindfulness has emerged as a dominant approach for improving practitioners’ mental health and wellbeing (29). Additionally, online self-care interventions have proven effective in improving social workers’ mental wellbeing, offering benefits in terms of cost-effectiveness and efficiency (25).

Despite these strategies suggested for individual implementation, exceptions exist in the literature that emphasize the need for collective responsibility (16). This perspective is reflected in emerging literature that challenges individualized notions of self-care. Profitt’s study examining social workers in Costa Rica who address gender-based violence advocates a critical and collective framework for self-care that acknowledges intersecting social and economic factors (30). Her research challenges individualized approaches to self-care as insufficient for capturing social work’s complexity as a profession focused on social change. She argues that effective self-care has to incorporate both the elements of professional practice while recognizing the developmental processes of awareness-building and professional challenges that social workers get throughout their careers. Grise-Owens et al. further advocate for incorporating ‘organizational wellness’ into self-care in social work to promote the idea that it is both a collective responsibility and an individual one (31). Only through this multi-level approach can social workers’ vulnerability be adequately addressed and their capacity for effective service maintained.

While research on self-care in social work has grown, current literature suggests a need for a more thorough examination of how social workers implement and experience self-care strategies in their daily professional and personal contexts. The effectiveness of self-care practices varies among practitioners with diverse needs, suggesting the importance of individualized approaches while highlighting implementation challenges (26). Research also suggests that successful self-care implementation requires a multi-level framework, encompassing both individual commitment and organizational support to foster environments conducive to practitioner wellbeing (32). However, research exploring barriers to self-care implementation remains in its early stages. Recent studies have begun addressing this imbalance by identifying obstacles at multiple levels that can undermine practitioners’ wellbeing efforts (65). The development and validation of a new scale, the Barriers to Professional Self-Care Scale (BPS-CS), represents an advancement in this area, also highlighting that effective self-care requires addressing both individual misconceptions and structural workplace factors rather than placing responsibility solely on practitioners (66). These complexities point to the value of in-depth explorations to better understand the nuances of self-care efficacy in practice.

In Scotland, efforts to explore and institutionalize self-care practices are underway, and the emerging evidence, particularly for mindfulness, is showing effectiveness with social work students (6, 33, 34). A further funded project nearing completion is looking at the experiences of social work students in Scotland concerning views and approaches toward self-care (16). The present study extends this line of inquiry by investigating self-care practices among qualified social workers in Scotland, addressing an important gap in the literature. Given the reported variations in self-care awareness and implementation across different countries and regions (25), this study provides valuable evidence that may inform related activity within the broader international social work context.

### The theoretical framework: vulnerability and mental health

Vulnerability theory, as conceptualized by Fineman, posits that vulnerability is both ‘universal and constant’ and ‘inherent in the human condition’ (35). This theoretical lens helps explain how helping professionals’ identity and working conditions create vulnerability that impacts their mental health. The theory suggests that vulnerability manifests differently based on individuals’ embodied and embedded positions within their social environment while emphasizing that institutions themselves can be vulnerable (36).

This theoretical perspective is also relevant for understanding social workers’ vulnerability. It challenges the traditional notion of helping professionals as purely resilient caregivers, acknowledging that they also experience inherent vulnerabilities through their work. Vulnerability has been recognized through multiple aspects with interactions among different vulnerability mechanisms that influence mental health outcomes (1). It also emphasizes that vulnerability operates at both individual and institutional levels, suggesting effective responses, including self-care, to be taken for both personal and systemic dimensions (24, 37).

While vulnerability theory explains the inherent and universal nature of vulnerability in helping professions, it addresses the importance of resilience-building through institutional support (33, 38). Within this framework, self-care emerges as an important response to these professional vulnerabilities, supporting practitioners in protecting their mental health while dealing with challenging circumstances. However, purely individualistic approaches to resilience-building may be insufficient and could perpetuate neoliberal tendencies to place responsibility solely on individuals (36). Instead, this theoretical approach suggests the need for more comprehensive, systemic responses that address both personal and institutional dimensions of practitioner vulnerability and stress.

Research documenting public health providers’ experiences with anxiety, fear, and professional distress has helped illuminate common mechanisms through which occupational demands may affect mental health and wellbeing (39, 40). Understanding these vulnerability mechanisms appears crucial for practitioners’ professional development and for maintaining quality service delivery (41, 42). The implications extend beyond social work to other helping professions, where practitioner wellbeing directly influences the effectiveness of healthcare system.

### Goals of the research

To address these research gaps and contribute to the growing body of knowledge on social worker wellbeing, this study employs a qualitative approach to explore four research questions: (1) How do social workers in Scotland currently implement self-care practices in their professional lives? (2) What personal and structural factors may influence social workers’ engagement with self-care practices? (3) What factors contribute to the effectiveness of self-care practices in supporting social workers’ mental health and wellbeing? (4) What implications might these findings have for enhancing social worker wellbeing in diverse global contexts?

## Methods

### Research design

This study employed a qualitative research method using semi-structured interviews to explore social workers’ personal experiences of self-care practices. A qualitative approach was chosen because it allowed for in-depth exploration of participants’ lived experiences and meanings attributed to self-care practices (6, 27, 33). Semi-structured interviews were selected as they provided flexibility to explore emerging themes while maintaining consistency across interviews (43).

Participants were recruited through email invitations sent to three local authorities in Scotland. The selection of these authorities was based on their diverse geographical locations and socio-economic contexts, allowing for variation in social workers’ experiences. Participation was voluntary, with the requirement of signed consent forms. Interviews were conducted between October 2023 and March 2024, accommodating participants’ preferences for online interviews via Teams for convenience.

The decision to conduct interviews online was based on its practical advantages, including time efficiency, ease of scheduling, and ensuring privacy conducive to open and candid sharing of experiences (44). In addition, online interviews have proved as effective as in-person interviews in terms of rapport building and data quality, thus maintaining the rigor of qualitative research (45).

Semi-structured interviews, lasting about 45 min on average, were designed with a comprehensive set of questions. The interview guide was developed based on existing literature on self-care practices and vulnerability theory. These questions explored participants’ interpretations of self-care, frequency of engagement in self-care activities, specific strategies employed, and the impact of self-care on both personal and professional aspects of their lives. The interviews also asked about challenges faced in adopting self-care practices, factors facilitating self-care promotion, external support needed for sustaining self-care, and expectations for future developments in self-care practices. For example, to understand how professional vulnerability shaped self-care practices, participants were asked, “What are the major challenges that you currently face with stress as a social worker?” and “How do you interpret the concept of self-care?” To explore the potential challenges for implementation, questions such as “Do your work surroundings and employing organization support the conduction of self-care?” and “What organizational barriers to self-care exist?” were asked. Questions about collective approaches, including “What support do you need to better implement self-care strategies?” and “What would be an ideal situation that you can imagine for your self-care?” were also incorporated. The latter question focused on exploring institutional support needs and organizational resources for self-care implementation. Additionally, cultural influences were examined through questions about participants’ backgrounds and how these shaped their views on self-care to capture diverse perspectives from international participants.

### Participants

Nine social workers participated in the final study, comprising eight females and one male from Britain, Canada, Switzerland, and China. Participants ranged in age from 29 to 69 years, with most between 30 and 40 years. Their educational backgrounds spanned undergraduate to graduate levels. As the study focused on frontline practitioners rather than managers or researchers, all participants were practicing social workers without doctoral qualifications. They worked across various areas, including children and families, justice services, and educational settings. Table 1 presents detailed participant demographics.

**Table 1:** Demographic characteristics of the participants (*N* = 9).

Demographic characteristic	Participants
Gender	
Male	1
Female	8
Age (29–69)	
<30	1
30–40	4
41–50	2
51–60	1
61–70	1
Education	
Bachelor of Social Work	3
Master of Social Work	6
Nationality	
British (including Scottish)	6
Canadian	1
Swiss	1
Chinese	1
Working location	
Edinburgh	7
Motherwell	1
Argyll and Bute	1
Work settings	
Children and Families	4
Criminal Justice	2
Community Justice	2
Educational Practice	1
Years of professional practice	
<3	3
3–10	4
>10	2
Diagnosis of mental health disorders after being social workers	
None	7
Depression	1
Anxiety	1

While the sample size was relatively small (*n* = 9), the final interviews showed recurring patterns in key themes, though additional perspectives might emerge with a larger sample. This level of data sufficiency aligned with the study’s exploratory aims to understand social workers’ self-care experiences while acknowledging that further research with larger samples could reveal additional insights.

### Data collection and analysis

All interviews were audio recorded with consent and transcribed using NVivo software. The research dataset comprised interview transcripts, researcher notes, consent forms, and demographic questionnaires. Transcript accuracy was verified through a thorough review by the first author.

Analysis followed Braun and Clarke’s thematic analysis framework, progressing through data familiarization, initial coding, theme identification, review, refinement, and final reporting (46). The process generated approximately 166 initial codes using both inductive and deductive approaches, allowing themes to emerge naturally while also considering vulnerability theory concepts. These initial codes were subsequently collated into potential themes (such as grouping “workload pressure,” “boundary challenges,” and “organizational demands” under “The Implementation Paradox”). The first author led this initial coding and pattern identification, with the second author independently reviewing these analyses to ensure rigor. Table 2 provides representative examples of these initial codes and how they were organized into themes, demonstrating the progression from coding to theme development.

**Table 2:** Example of thematic analysis process.

Themes	Description	Sub-themes	Initial codes (representative)
Understanding and conceptualizing self-care	How social workers understand, define, and conceptualize self-care in their personal and professional lives	Broad and individualized nature	massive term, fluid, covers a lot of things, individualized, different with ages
		Personal wellbeing	looking after yourself, keeping yourself well, put yourself first, protected opportunity to focus on what your needs are
		Professional effectiveness	better work for clients, rich communication with clients, self-confidence of work ability
		COVID-19 impact	changes about awareness after COVID-19, read more about work-life balance
		Self-care activities	mindfulness, work-life balance, exercise, reading, meditation, time with loved ones
The implementation paradox	The tensions and challenges social workers face when attempting to implement self-care practices	Workload challenges	writing reports, running clients, not enough time, exhaustion
		Boundary challenges	overworking problem, work overtime, responsible for clients, feeling of guilty
		Organizational expectations vs. reality	organizationally expectations, want to meet the requirements, insufficient resources
		Emotional labor	emotional labor, vicarious trauma, traumatized by client experiences, taking on other’s experience
		Professional culture	social work profession stereotype of overworking, peer pressure, self-sacrifice
Toward sustainable self-care practices	Pathways and expectations toward sustainable self-care	Intentional action and boundaries	good planning, confidence to say no, set self-boundaries, self-awareness
		Supportive leadership and management	atmosphere of caring and valuing wellbeing, positive and open communication, flexible working
		Individual responsibility	identity the personal needs, look for the support by self, continuous practices
		Educational preparation	self-care training or education, recognition, practical activities
		Cultural perspectives	cultural element, breakdown the culture of professional demands

As the process continued, themes were reviewed against coded extracts and the entire dataset to ensure coherence, with the full research team discussing any discrepancies until consensus was reached. Themes were then refined and clearly defined through collaborative team discussions to capture the data’s essence while maintaining theoretical alignment. The final analysis was written with compelling extract examples to demonstrate theme prevalence and significance. This collaborative analytical approach aligned with the study’s aim to understand how self-care is conceptualized and implemented, including individual and structural barriers to practice.

### Ethics

The study received ethical approval from the author’s University, where the above-mentioned self-care with students’ project is hosted. Participants received detailed information sheets outlining study purposes, procedures, and their rights. Written informed consent was obtained prior to participation, with explicit permission for audio recording and data use. Data confidentiality was maintained through pseudonymization and secure storage of all research materials.

## Findings

Through systematic thematic analysis of the interview data, three overarching themes emerged that illuminate the complex dynamics of self-care among social workers in Scotland: (1) Understanding and Conceptualizing Self-Care, (2) The Implementation Paradox, and (3) Toward Sustainable Self-Care Practice. These themes revealed a progression from basic conceptualization through practical challenges to potential pathways for sustainable implementation. The professional background information of the participants interviewed is presented in Table 3.

**Table 3:** Professional background of interview participants (*N* = 9).

Participant ID	Nationality	Work settings	Years of social work practice
Participant 1	British	Educational Practice	>10
Participant 2	British	Children and Families	<3
Participant 3	Canadian	Criminal Justice	3–10
Participant 4	British	Children and Families	3–10
Participant 5	Swiss	Community Justice	<3
Participant 6	British	Criminal Justice	3–10
Participant 7	British	Community Justice	<3
Participant 8	Chinese	Children and Families	3–10
Participant 9	British	Children and Families	>10

### Theme 1: understanding and conceptualizing self-care

As articulated by participants, self-care represents a multifaceted, individualized concept that extends beyond mere stress relief, integrating both personal wellbeing and professional effectiveness, with understanding and practices notably evolving since COVID-19.

Participants with either much or less knowledge of self-care all presented self-care as highly personal practice encompassing a broad range of activities tailored to individual needs and preferences:

*Self-care is a very individual thing, and everybody will need different levels of self-care (Participant 1).*

*Self-care is just a massive term to me… Good supervision is part of self-care… But then also what you’re doing when you’re finished with work and you decompressing… the kind of more active things that you are doing… (Participant 2).*

While recognizing self-care’s primary purpose as “looking after self” and “keeping self well,” all participants invariably linked its ultimate benefits as enhancing their professional performance and effectively working with service users:

*You have to put yourself first to be an effective worker (Participant 1).*

*Self-care is really about looking after yourself and keeping yourself well… to be able to make sure that you’re effectively able to meet the needs of our clients (Participant 3).*

The connection between self-care and professional practice was particularly evident in discussions about client interactions:

*It’s really important for working with the families and young people, [so] you need to be able to look after yourself and make sure that you can do your job properly (Participant 4).*

*We’re trying to be very conscious of our responses to someone so that we create a comfortable environment for them rather than a judgmental one. And I think that takes up a lot of energy in a way that not all jobs do. So, I guess, self-care is a way to kind of recharge that energy (Participant 5).*

The understanding and practice of self-care among social workers have evolved markedly since COVID-19. Four participants spontaneously reported experiencing “more stressed” and “less supported” while working from home, which prompted increased attention to self-care practices:

*[I] read more about trying to find that work-life balance and things like that…I think there’s always space for trying different things (Participant 2).*

However, this enhanced understanding has not necessarily translated into effective implementation for all the workers. This was evident in one participant’s struggles to apply self-care principles to themselves:

*It’s a concept I talk to my clients about a lot, but not fully a concept that I probably talk to myself about (Participant 5).*

Two participants thus responded by advocating for a more natural, less formalized approach to self-care into their daily routines rather than treating it as ‘extra’ or additional pressure:

*The most important one is probably a bit controversial. It’s just being mindless escapism (Participant 1).*

*My instinct has been to not overthink self-care and do the things I would usually, normally, I would find relaxing, or sort of routine related to self-care (Participant 7).*

### Theme 2: the implementation paradox

This theme explores the fundamental tensions and challenges social workers face when attempting to implement self-care practices. These challenges manifest at personal, professional, and organizational levels, creating a complex web of barriers to effective self-care implementation.

The exercises of self-care have been varied among workers to effectively manage stress and other occupational hazards, including vicarious trauma (16, 24).

*I’ve done breathing exercises; I’ve done visualizations with clients. And when even if I’m guiding it, I can feel the parts of my body kind of coming down… the way it kind of helps my body trick my brain a little bit into like slowing down and getting out of kind of the survival mode that I might be in (Participant 5).*

*I think for me when the workday is over, I need to have something [for self-care] … like something to kind of bridge between work and then and my personal life in the evening start in but quite often I find even like whether it’s going to a gym class or walking home right after work and just having that time where I’m actively doing something (Participant 6).*

However, implementation remained challenging. A primary challenge emerged in balancing work demands with personal needs. The demanding nature of the profession makes it challenging for individuals to prioritize their wellbeing amidst competing demands;

*I think the [work] demand is just too high. We just try to keep up with the demand and put ourselves at the bottom of the list… So, self-care is not something being sort of acknowledged (Participant 8).*

The challenge of maintaining boundaries was particularly evident in participants’ struggles with work hours and responsibilities, pushing the worker to accept that using personal time to complete work tasks was unavoidable:

*I try to put boundaries in place. But equally, when there are lots of court reports coming in, at one point you want to support your team (Participant 6).*

*I think there’s something about knowing that if you don’t do a thing, it’s a person who suffers on the other end… it like it feel there’s much more pressure there to achieve things even though for me it like it takes like 10 minutes of my day, it could be really important to this other person. And so, I think it’s harder to set boundaries about not working overtime. (Participant 5).*

This challenge was intensified in the context of increased remote working since COVID-19:

*I think when I’m working from home [since COVID-19], it’s harder for me to keep a boundary cause the laptops there, the telephones there (Participant 3).*

Participant 9 thus discussed the persistent tension between recognizing personal needs and meeting professional demands after 20 years of experience as a social worker. Her reflections encompassed not only her personal experience but also stories from fellow practitioners, with a common paradox in the profession in which the emotional investment in service users often conflicts with self-care practices. Moreover, this participant highlighted the systemic pressures that exacerbate this paradox and organizational expectations to make more changes:

*The system and the organization have a responsibility to try and make this job less stressful for people and more manageable (Participant 9).*

Despite the expectations for organizational support, the data then reveals a gulf between organizational rhetoric and practical support for self-care practices since participants believed that although organizations demonstrated a stated commitment to self-care, actual implementation remains limited, with insufficient resources allocated to address their vulnerability:

*My organization wants us to do self-care, but I think there’s a difference between them wanting us to do self-care and then really facilitating it, right? …They want us to do self-care, but not proactively enough to truly enable us to do self-care…it’s hard to form habits within a work environment that has so many demands (Participant 5).*

Further amplifying this institutional disconnect, the accounts from social workers in this study suggested that support and resources provided by social work institutions or systems for self-care appeared more “theoretical” than “practical” since there was a lingering expectation that “jack-of-all-trades” social workers would resolve issues independently:

*So, they’ll [social work management] say the right things, but when it comes down to it, they want a worker who has a significant level of professional resilience just to get almost all the work [done]…You can’t rely on management or your organization to support you (Participant 1).*

The professional culture further complicated self-care implementation, with participants recognizing the role prevailing cultures play and advocating a shift toward making self-care visible and accepted:

*I suppose seeing other people doing stuff, like sort of the culture within the teams, is a barrier (Participant 4).*

*You’ve heard me in this interview talk about how the demands of the job take over ultimately. And unfortunately, I think that’s a bit of a cultural thing that’s in social work, and I think there needs to be something to break the cycle (Participant 6).*

### Theme 3: toward sustainable self-care practice

This theme explores pathways toward sustainable self-care through the integration of individual strategies, organizational support, and systemic changes. The findings emphasize a shift from individual coping mechanisms toward collective resilience-building approaches.

Experienced practitioners emphasized the importance of intentional action and boundary-setting:

*I think that’s probably been the most helpful for me, creating a space where I can spend time processing what’s happened before I get into my home (Participant 3).*

*I’m trying to practice self-care as a habit rather than, oh, I feel stressed, I need to do this thing to self-care. It does kind of give me the sense of feeling at ease and balanced…I have a hobby…and I prioritize them (Participant 9).*

The responsibility of social workers in seeking external resources by themselves to promote their wellbeing was highlighted:

*It was a week of wellbeing activities that they [management] published… people [other social workers] might not have even been aware of that…I think with the institution on a wider level, you have to look a little bit for it, and it’s very much on you to do that and to find that support (Participant 1).*

Establishing and maintaining boundaries emerged as another crucial aspect of promoting self-care. Two social workers with longer years of working experience tended to have greater levels of comfort about expressing their needs in this way and achieving better wellbeing:

*You must set the boundaries; you have to know the limit (Participant 8).*

*Just the confidence to say, no, I didn’t have time for that. I did not work more than my hours this week, and that’s okay (Participant 9).*

Effective leadership and supportive management emerged as crucial facilitators:

*And I have a fantastic team leader…We’ve always done supervision in a fairly kind set way…She’ll ask about my wellbeing, what’s going on? And she doesn’t just mean work, she’ll say, how is your personal life going? We have team-away days as well, which is a nice little thing in our team (Participant 2).*

*I think having a really good manager who gets it is key…we have an agenda for supervision, and always at the top of the agenda is wellbeing and how we’re doing. And that’s where we’d be able to bring up any concerns about the impact fact of the work or what I’m doing to kind of keep well (Participant 3).*

This recognition of shared responsibility between practitioners and management was articulated by Participant 9:

*I think one of my self-care strategies is to be much less isolated with the responsibility and to feel very clearly that my employer has a responsibility here and some of the pressure is from the perceptions of social work (Participant 9).*

Beyond immediate workplace support, participants identified educational preparation as crucial for developing sustainable self-care practices. Their reflections highlighted gaps in current educational approaches while suggesting practical improvements:

*Students understand the importance of having good coping mechanisms and a work-life balance, but I don’t think they fully appreciate the level of stress that will come their way once they’re qualified. And I think it’s only when you get to that point that you then really need to reflect on self-care and realize, no, I need to pay attention to this (Participant 1).*

The importance of practical, experiential learning was addressed by two other participants as they expressed a need for integrating hands-on self-care activities into the curriculum, providing opportunities for social workers to develop practical skills for managing stress and promoting wellbeing:

*Practical activities [should be considered] for integrating into the speech…. they [the educators] also should be engaging in the activities as part of the education (Participant 5).*

*I think training does need to be regular, and it doesn’t necessarily need to be like this formal thing. It can be just, you know, like an informal development session (Participant 3).*

Cultural perspectives were found to potentially influence the understanding and implementation of self-care practices. While all eight participants from Western cultural backgrounds discussed their self-care needs and implementation, one participant from Eastern culture shared the experience of lacking knowledge and practice due to cultural views:

*It’s (self-care) not part of the training that we received…And I think it’s also that cultural element coming from the Chinese culture, whenever we talk about self, it always looks like selfish (Participant 8).*

As such, when asked “What would be an ideal situation that you can imagine for your self-care,” participants emphasized the need for tangible institutional support to enable direct engagement with self-care, such as flexible work schedules, designated time and space for self-care activities during work hours, and access to mindfulness and wellness programs:

*I think obviously, the quality of supervision and having a like manager who has a genuine interest in your welfare is important (Participant 6).*

*You need your employer to offer things like sessions that are going to take you out of your comfort zone and offer you some new types of things like chair yoga… But I think they also need to support you when you say that you’ve had enough when you say, and I need a break, I’m at burnout point and I feel stressed. You need the space to do that (Participant 2).*

## Discussion

Informed by the views and lived experience of a small group of social workers in Scotland, this study has examined the implications of currently adopted self-care strategies and their potential to enhance the wellbeing of practitioners. Guided by vulnerability theory, this analysis reveals that self-care operates at both individual and institutional levels while being shaped by broader systemic and cultural factors among social workers. Although individual efforts play a crucial role in implementing self-care, the study emphasizes the need for systemic support and multi-level interventions for the long-term support of social workers, which is essential to sustain their wellbeing and the quality of their work with service users. Consequently, a combination of educational, organizational, and systemic initiatives is required to foster an environment in which social worker self-care is embedded in the fabric of the profession, rather than at present where it appears to be addressed in an *ad hoc* or tokenistic fashion and seen largely as the responsibility of individual practitioners.

### The individual-professional interface in self-care practice

This study reveals how professional vulnerability fundamentally shapes social workers’ understanding and implementation of self-care practices. As Fineman’s vulnerability theory suggests, vulnerability manifests differently based on individuals’ embedded positions within their social environment (35). The participants demonstrate this through their individualized interpretations and approaches to self-care, reflecting how professional vulnerability creates unique challenges in balancing personal wellbeing with work demands.

The effectiveness of self-care is evidenced through practitioners’ enhanced capacity for service delivery despite facing personal and systemic barriers. Research findings demonstrate a critical link between self-care practices and the quality of engagement with service users, supporting previous studies (8, 9). Effective self-care leads to significant improvements in practitioners’ mental wellbeing, including greater self-compassion, self-efficacy, and resilience (47, 48), ultimately supporting more sustainable and empathetic interactions with service users (10).

Finding a balance between proactive effort and natural integration of self-care into daily routines is found crucial to prevent these practices from becoming additional burdens. Incorporating self-care into personal hobbies and making it part of a routine appeared to help social workers achieve relaxation and mitigate stress more effectively. However, participants were emphatic about the limitations of relying solely on individual resilience without systemic support. The analysis revealed that social workers experience vulnerability in ways that directly impact their self-care practices. Participants consistently described a fundamental tension between acknowledging their own vulnerability, including their need for self-care, and meeting intense professional demands. This tension exemplifies what the vulnerability theory describes as the complex interplay between embodied experience and institutional contexts (36).

### Systemic and cultural influences on self-care implementation

As a key public health provider, social workers’ personal wellbeing and service provision are significantly influenced by broader systemic factors, particularly the prevailing neoliberal management approaches in social services (48). Participants’ experiences reflect how market-driven efficiency measures and increased accountability demands create systemic barriers to effective self-care. The emphasis on measurable outcomes and cost-effectiveness often overshadows the need for practitioner wellbeing, as evidenced by participants’ struggles with workload management and resource allocation. As vulnerability theory suggests, this institutional failure to adequately respond to professional vulnerability creates additional layers of precarity for practitioners (35, 36).

Systemic barriers appear in multiple ways. While organizational support is recognized as crucial for fostering self-care among social workers (26, 31), the participants revealed a significant gap between organizational rhetoric and actual support. This gap highlights deeper systemic issues where organizations verbally acknowledge professional vulnerability but fail to provide the institutional resources necessary for resilience. Other factors, such as heavy workloads, long hours, and limited supervision, further complicate self-care practices and contribute to inconsistencies in their implementation for either personal wellbeing or professional improvement (49, 50).

Even though the relevant data was limited in scope, one participant pointed out that cultural differences highlight important considerations for future research. While work demands and the need for stress reduction are universal, cultural attitudes toward self-care differ. This participant from a Confucian background expressed a sense of shame in prioritizing self-care, viewing it as selfish, which aligns with values of self-sacrifice (51). This reflects how cultural frameworks shape the conceptualization and implementation of self-care. Building on these insights and current practices in Scotland, our research team has initiated cross-cultural explorations of self-care implementation, particularly in East Asian contexts (25). These efforts recognize the importance of cultural sensitivity in developing effective self-care approaches rather than simply adopting standardized Western models. The ongoing work in China, for example, seeks to understand how self-care concepts and practices can be meaningfully adapted to local cultural contexts while maintaining their fundamental benefits for practitioner wellbeing (19). This emerging cross-cultural dialogue presents opportunities for developing more nuanced and culturally responsive approaches to social worker self-care, with findings from these international investigations planned for future publication.

The COVID-19 pandemic has further exposed and exacerbated these systemic challenges (24, 25). Remote working arrangements, while necessary for public health, have intensified the blurring of professional and personal boundaries. This demonstrates how broader societal changes can create new vulnerabilities and challenges for self-care implementation, requiring adaptive responses at both systemic and cultural levels.

### The importance of collective approaches in self-care

Building on existing literature that highlights the tension between the ethical responsibility for self-care and the professional expectation to prioritize others’ needs (52), this study emphasizes the importance of organizational support in fostering collective self-care. Participants questioned the effectiveness of individualistic self-care approaches within the current working environment and expressed a desire for more collective strategies. These approaches challenge the individualistic focus often associated with self-care and underscore the need for collective action to address broader systemic challenges. Creating supportive team environments, encouraging open discussions, promoting peer support networks, and engaging in communal activities outside of work were seen as vital and increasingly necessary to confront cultural norms that promote isolation and individual responsibility for wellbeing (16).

The findings advocate for a paradigm shift in how self-care is conceptualized and implemented within social work education and practice. Previous research advocates the importance of a collective approach, where organizational responsibility plays a crucial role in creating a supportive environment (26, 31). This foundation has been strengthened by recent empirical studies that offer more comprehensive frameworks. Cole et al. (53) advanced this discourse by proposing a new conceptual framework that transforms self-care from an individual necessity to a shared organizational responsibility, identifying three interconnected domains of strategies. Jian et al.’s systematic review expanded the scope further by addressing self-care as a global issue transcending geographic, cultural, and professional boundaries, highlighting implementation across regions and practice settings (24). This study reinforces the need for integrating self-care into the core values of the profession, redefining it as an essential aspect of both personal and professional life rather than an isolated responsibility. However, there exists a significant gap between ethical guidelines, as outlined in the Global Social Work Statement of Ethical Principles (54), and real-world application (55).

To bridge this gap, this study supports systematically integrating self-care training into social work curricula. Aligned with previous studies (31, 56), participants emphasized the need for establishing an early professional foundation for practitioners to prioritize wellbeing and achieve holistic growth. While existing literature acknowledges the importance of balancing personal and professional responsibilities (57), current training programs were reported as inadequately addressing these principles. This study provides evidence that practical, hands-on training and regular development sessions are essential for equipping social workers with actionable strategies (31). By embedding these practices into education and ongoing professional development, training programs can better prepare practitioners to manage stress and sustain their wellbeing throughout their careers.

### Implications for public health

The research on social workers and their practical activities helpfully illuminates important considerations for public health policy and healthcare workforce sustainability more generally (58). As this study reveals that practitioner wellbeing fundamentally impacts not only population health outcomes but also healthcare service delivery, several suggestions for broader public health policy and practice have been considered.

At the healthcare system level, there is still an urgent need for structural reforms in workforce health protection (59). Healthcare institutions are recommended to develop comprehensive workplace wellness programs that address both individual and systemic factors affecting practitioners’ wellbeing. This includes establishing clear policies for workload management, implementing regular supervision and support mechanisms, and allocating dedicated resources for staff wellbeing initiatives. Such organizational-level interventions are crucial for preventing burnout and maintaining workforce stability, which directly impacts service quality and public health outcomes.

This study reveals implications for health policy development. Current neoliberal management approaches in healthcare services often prioritize measurable outcomes and cost-effectiveness over practitioner wellbeing (60). This suggests a need for policy reforms that recognize practitioner wellbeing as integral to service quality. Effective policies should mandate minimum standards for workplace wellness programs and establish clear guidelines for workload management and resource allocation. Additionally, wellbeing metrics should be integrated into healthcare service evaluation frameworks and supported by dedicated funding mechanisms for implementing collective self-care initiatives. These policy changes also need to consider cultural competency in workforce support programs, acknowledging the diverse needs of healthcare practitioners.

This finding highlights the need for sustainable attention to the healthcare workforce’s wellbeing in the context of global health challenges (61, 62). The COVID-19 pandemic exposed vulnerabilities in healthcare systems and reinforced the critical importance of supporting frontline workers, as evidenced by the participants’ experiences. While the physical impact of the crisis has diminished, concerns and risks among public health providers remain evident, particularly as necessary protections have not been thoroughly implemented for future risk prevention (63). The persistence of these challenges suggests a gap between crisis response and long-term organizational change. Healthcare organizations are thus advised to develop resilient systems that can adapt to emerging public health challenges while maintaining workforce wellbeing. This includes creating flexible working arrangements, establishing emergency support protocols, and building robust peer support networks for long-term workforce sustainability.

### Limitations

This study has several limitations. The relatively small and geographically constrained sample (*n* = 9 social workers from three local authorities in Scotland) may limit the generalizability of current findings to broader contexts. Furthermore, participants varied significantly in their prior knowledge, training, and experience with self-care practices, which could have influenced their perspectives and responses. Given the qualitative nature of the study, generalizability in terms of statistical representativeness was not the primary goal of this study; instead, the goal was to achieve depth and richness in understanding social workers’ experiences and perceptions of self-care, guided by vulnerability theory. However, the research team monitored the recurrence of themes during the analysis and compared newly identified codes with those from earlier interviews to establish the key patterns when minimal new themes emerged. While this study offers meaningful insights, future research with a larger, more diverse sample is recommended for broader generalizability and a more exhaustive exploration of self-care practices among social workers (64). Additionally, while work-life boundary challenges emerged in the findings, this study did not explore theoretical perspectives that frame these boundaries as inherently blurred rather than distinct. Future research could examine how such conceptualizations might help social workers develop more realistic expectations when navigating professional and personal domains. Despite these limitations, this study provides valuable qualitative insights that can inform policy and practice improvements, benefiting practitioners, educators, and public health institutions seeking to promote sustainable self-care solutions.

## Conclusion

Through thematic analysis of interviews with nine Scottish social workers, this study reveals how self-care practices in social work are fundamentally shaped by professional vulnerability and require robust institutional responses. The findings underscore that effective self-care among social workers cannot rely solely on individualized approaches; instead, it requires systematic engagement and robust support from organizational and policy levels. By illuminating the paradox of self-care implementation, where practitioners recognize the necessity of self-care yet encounter substantial systemic barriers, the study advocates shifting the discourse from purely individual responsibility toward comprehensive institutional responses. Concrete structural and policy-level changes, including workload management reforms, supportive supervision practices, culturally sensitive training, and accessible workplace wellness resources, are essential for building collective resilience within the workforce. Ultimately, prioritizing practitioner wellbeing as a fundamental public health concern holds significant implications not only for social workers but for broader healthcare workforce sustainability and the quality of public health service delivery.

## Data Availability

The raw data supporting the conclusions of this article will be made available by the authors without undue reservation.
